# Efficacy of Recombinant Canine Distemper Virus Expressing *Leishmania* Antigen against *Leishmania* Challenge in Dogs

**DOI:** 10.1371/journal.pntd.0003914

**Published:** 2015-07-10

**Authors:** Ryuichi Miura, Takanori Kooriyama, Misako Yoneda, Akiko Takenaka, Miho Doki, Yasuyuki Goto, Chizu Sanjoba, Yasuyuki Endo, Tomoko Fujiyuki, Akihiro Sugai, Kyoko Tsukiyama-Kohara, Yoshitsugu Matsumoto, Hiroki Sato, Chieko Kai

**Affiliations:** 1 Laboratory Animal Research Center, Institute of Medical Science, The University of Tokyo, Tokyo, Japan; 2 Department of Molecular Immunology, School of Agriculture and Life Sciences, The University of Tokyo, Tokyo, Japan; 3 Joint Faculty of Veterinary Medicine, Kagoshima University, Kagoshima, Japan; Universidade Federal de Minas Gerais, BRAZIL

## Abstract

Canine distemper virus (CDV) vaccination confers long-term protection against CDV reinfection. To investigate the utility of CDV as a polyvalent vaccine vector for *Leishmania*, we generated recombinant CDVs, based on an avirulent Yanaka strain, that expressed *Leishmania* antigens: LACK, TSA, or LmSTI1 (rCDV–LACK, rCDV–TSA, and rCDV–LmSTI1, respectively). Dogs immunized with rCDV-LACK were protected against challenge with lethal doses of virulent CDV, in the same way as the parental Yanaka strain. To evaluate the protective effects of the recombinant CDVs against cutaneous leishmaniasis in dogs, dogs were immunized with one recombinant CDV or a cocktail of three recombinant CDVs, before intradermal challenge (in the ears) with infective-stage promastigotes of *Leishmania major*. Unvaccinated dogs showed increased nodules with ulcer formation after 3 weeks, whereas dogs immunized with rCDV–LACK showed markedly smaller nodules without ulceration. Although the rCDV–TSA- and rCDV–LmSTI1-immunized dogs showed little protection against *L*. *major*, the cocktail of three recombinant CDVs more effectively suppressed the progression of nodule formation than immunization with rCDV–LACK alone. These results indicate that recombinant CDV is suitable for use as a polyvalent live attenuated vaccine for protection against both CDV and *L*. *major* infections in dogs.

## Author Summary

More than 1 million new cases of leishmaniasis occur throughout the world every year. Leishmaniasis typically presents as one of two clinical forms, either cutaneous or visceral. Dogs harboring *Leishmania* act as reservoirs, and are closely associated with human infections in South America and southern Europe. Therefore, immunization of dogs with effective vaccines against *Leishmania* will also effectively prevent *Leishmania* infection in humans. In this study, we have evaluated the utility of recombinant canine distemper viruses (CDVs) that express *Leishmania* antigen as effective polyvalent candidate vaccines against CDV and cutaneous *Leishmania* infections. The results indicated that recombinant CDV completely protected against challenge with a virulent strain of CDV. Furthermore, mixed immunization with three recombinant CDVs that express different antigens that mediate distinct immune responses, significantly reduced the nodule size after *Leishmania major* challenge. These results strongly suggest that a cocktail of multiple antigens confers more effective immunity throughout the life cycle of *Leishmania*. We propose that a combination of recombinant CDV-based vaccines expressing different antigens has utility as a polyvalent vaccine for the prevention of leishmaniasis epidemics by inhibiting the transmission of the parasites through dogs.

## Introduction

Leishmaniasis is a major infectious disease caused by the parasitic protozoan *Leishmania* in both humans and dogs. It occurs across 88 countries and affects 12 million people in tropical and subtropical regions. The World Health Organization reported that in 1993, leishmaniasis was one of the six major tropical diseases in developing countries. Leishmaniasis is a complex disease with various symptoms, and includes cutaneous, mucocutaneous, and visceral forms, displaying a broad spectrum of zoonotic diseases in humans and animals [1]. More than 1 million new cases of leishmaniasis occur throughout the world every year, predominantly as the cutaneous form (along with one million cases of cutaneous leishmaniasis and 300,000 cases of visceral leishmaniasis) [2]. The parasites are naturally transmitted by blood-sucking sand flies among reservoir animals, including rodents and dogs, and are accidentally transmitted to humans by these animals.

Leishmaniasis in humans is caused by several species of *Leishamania*, which lead to strikingly different pathological responses. The cutaneous form of the disease, which is caused by species such as *L*. *major* and *L*. *tropica* accounts for more than 50% of new cases of leishmaniasis. It results in formation of skin ulcers at the site of the sand fly bite, usually on exposed parts of the body. The disease is most often self-limiting, but the time period to lesion resolution varies between species and between individuals. Visceral leishmaniasis, also known as kala-azar, is the most severe and often fatal form of the disease. Visceral species such as *L*. *donovani*, *L*. *infantum* and *L*. *chagasi*, target visceral organs and result in a pentad of syndromes comprising fever, weight loss, splenomegaly, hepatomegaly and anemia. Because of the lack of effective therapy, it is difficult to cure patients with late-stage infections.

In the case of canine leishmaniasis, clinical symptoms are varied and range from asymptomatic to fatal systemic disease. Dogs act as reservoirs of *L*.*infantum and L*. *chagasi* mainly, and others *L*. *tropica*, *L*. *major* and *L*. *brasiliensis*, and are closely associated with human infections in South America and southern Europe [3]. The elimination of canine leishmaniasis in Brazil correlated with a reduced prevalence of the disease in humans [4]. Therefore, treating dogs with effective vaccines against *Leishmania* will also effectively prevent *Leishmania* infection in humans [5].

Most studies of canine leishmaniasis have focused on the visceral form, with observations of both naturally and experimentally infected animals [6–9]. However, experimental models of canine cutaneous leishmaniasis are scarce, although the cutaneous form of the disease occurs in the majority of cases [10,11].

There is presently no vaccine against leishmaniasis, although extensive evidence from studies in animal models indicates that protection can be conferred by immunization with antigens (reviewed in [6–9]). A variety of different molecules have been tested, and some have shown protective activity in animal models, and vaccines against canine visceral leishmaniasis such as Leishmune (FML antigen), Leish-Tec (A2 antigen), Canileish (LieSap antigen), LbSap (*L*. *braziliensis* antigen) have previously been published.

Canine distemper (CD) is a lethal infectious disease of dogs and other members of the family Canidae, presenting as fever, pneumonia, bronchitis, leukopenia, severe diarrhea, and sometimes encephalitis [12]. Canine distemper virus (CDV), the causative agent, is a member of the family *Paramyxoviridae* and the genus *Morbillivirus*, which includes measles virus and rinderpest virus. Live attenuated CDV vaccines were developed and introduced in the 1950s, rapidly reducing the incidence of CD in dogs. However, CD outbreaks, even involving vaccinated dogs, have been reported worldwide since the 1990s [13–18]. implying that these vaccines are insufficiently efficacious to protect dogs against the currently circulating wild-type CDV strains.

We previously isolated a recently prevalent CDV strain, the Yanaka strain [19], that is avirulent in dogs and induces a high titer of neutralizing antibodies [20]. We demonstrated that dogs inoculated with CDV-Yanaka are completely protected against challenge with both old and recent virulent CDV strains [20], strongly suggesting that the Yanaka strain is a potential novel vaccine strain. We successfully established a reverse genetics system for CDV-Yanaka [21] that allows us to generate recombinant viruses expressing foreign genes. This technique can be used to develop new polyvalent vaccines based on CDV. CDV vaccination usually induces life-long immunity against CDV infection in dogs after a single injection. Therefore, a recombinant CDV (rCDV) carrying a foreign gene encoding a neutralizing epitope against a specific pathogen should induce long-term immunity against both CDV and the pathogen.

In the present study, we attempted to generate recombinant CDV-Yanaka expressing *Leishmania* antigens. We selected three protein antigens: LACK (*Leishmania* homologue for receptors of activated C kinase receptor), TSA (*L*. *major* homologue of eukaryotic thiol-specific antioxidant), and LmTSI1 (*L*. *major* homologue of eukaryotic stress-inducible protein 1). LACK, which is expressed throughout the *Leishmania* life cycle, has been extensively studied. Vaccination with either LACK DNA or LACK protein and interleukin 12 (IL-12) DNA induced long-term protection [6–9]. TSA was discovered by screening expression libraries to characterize the immune responses elicited by proteins isolated from filtrates of *L*. *major* promastigote cultures [22]. Immunizing BALB/c mice with recombinant TSA protein formulated with either IL-12 or TSA DNA induced strong cellular immune responses and conferred protective immunity against *L*. *major* infection [6–9,22,23]. LmSTI1 was identified when an *L*. *major* amastigote cDNA library was screened with sera from BALB/c mice infected with *L*. *major* [24]. Vaccination experiments with recombinant LmSTI1 protein plus either IL-12 or LmSTI1 DNA elicited a mixed cellular response that was skewed toward a T-helper 1 cell (Th1) phenotype, and protected BALB/c mice from infection [6–9,23,24]. In particular, it has been reported that immunization with a cocktail of *Leishmania* antigens confers greater protection against challenge than immunization with individual antigens [25,26]. The coadministration of TSA and LmSTI1 or a TSA–LmSTI1 fusion protein has been reported to enhance this protective immunity [6–9].

Based on our ability to generate rCDV and our knowledge of candidate vaccines against leishmaniasis, we generated rCDVs expressing *Leishmania* antigens and evaluated their efficacy as polyvalent vaccines against CDV and *Leishmania* infections.

## Methods

### Cells, viruses and parasite

Human embryonic kidney (HEK) 293 cells (RIKEN BioResource Center: RCB1637) were maintained in Dulbecco’s modified Eagle’s medium containing 5% fetal calf serum (FCS). B95a (marmoset lymphoblastoid) cells [27] were gifted from Dr. F. Kobune (National Institute of Health, Japan), and were maintained in RPMI1640 containing 5% FCS. The CDV-Yanaka strain [28] and rescued viruses were grown on B95a cells. The recombinant vaccinia virus, MVA–T7, which expresses T7 RNA polymerase, was a gift from Dr. T. Barrett and Dr. M Baron (Institute for Animal Health, UK). The virulent CDV strain, Snyder Hill, was passaged in dog brains, as described previously, and the brain homogenates were stored at −80°C [20]. Infective promastigotes of *L*. *major* strain PM2 were prepared as described previously [29]. In brief, promastigotes of *L*. *major* PM2 were maintained at 25°C in 199 medium (Nissui Pharmaceutical, Tokyo, Japan) containing 10% FCS and 25 mM HEPES, pH7.4. Late log-phase parasites were harvested and used in the experiments.

### Plasmid construction

**Fig 1:**
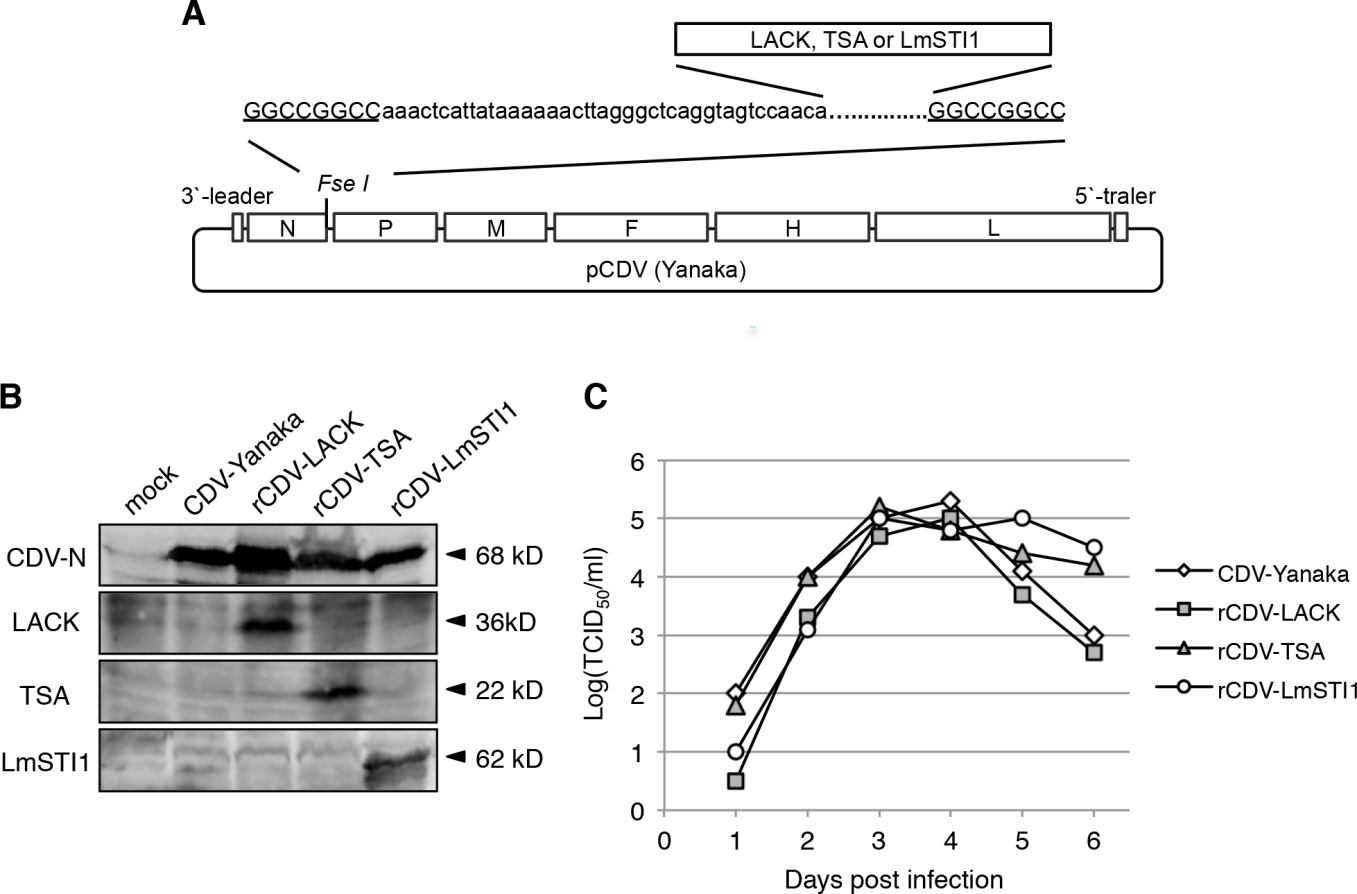
Generation and *in vitro* characterization of rCDVs. (A) Schematic model of pCDV (Yanaka) with the *Fse*I site introduced between the N and P genes. Each *Leishmania* antigen gene carrying the CDV transcription signal unit (lower case) and *Fse*I site (underlined) was inserted at the *Fse*I site. (B) The rescued recombinant viruses were identified by immunoblotting analysis. Cell lysates were examined with anti-CDV-N, anti-LACK, anti-TSA and anti-LmSTI1 antibodies. The bands corresponding to CDV-N (68 kDa), LACK (38 kDa), TSA (22 kDa) and LmSTI1 (62 kDa) are indicated by arrowheads. (C) Kinetics of recombinant viral growth. B95a cells were inoculated with a recombinant virus at a multiplicity of infection of 0.01, and then harvested on the indicated day. The titers of the viruses were determined using a TCID_50_ assay.

A full-length LACK cDNA was isolated from *L*. *donovani* [29]. The cDNAs for TSA and LmSTI1 were isolated from *L*. *major* [22,24]. After confirmation by sequencing, the cDNAs were reamplified by PCR using a forward primer containing the *Fse*I restriction site and the CDV transcription signal unit (aaactcattataaaaaacttagggctcaggtagtccaaca) at its 5’ end and a reverse primer containing the *Fse*I site at its 5’ end (Fig 1A). The amplified cDNA fragments were inserted into the *Fse*I site in pCDV(Yanaka), which encodes the full-length cDNA of the Yanaka strain RNA genome, previously established by our group [21] (Fig 1A).

### Viral rescue

CDV rescue was performed as described previously [21]. In brief, HEK293 cells infected with MVA–T7 were transfected with the full-genome plasmid described above, together with expression plasmids encoding viral nucleoprotein (N), phosphoprotein (P), and large protein (L) (pKS–N, pKS–P, and pGEM–L, respectively), using FuGENE6 Transfection Reagent (Invitrogen, Carlsbad, CA, USA). Two days later, the transfected HEK293 cells were overlain with B95a cells. Syncytia formed by the rescued viruses were observed approximately 3 days later. The viruses were harvested, and their titers determined with the limiting dilution method and expressed as the 50% tissue culture infective dose (TCID_50_).

### Preparation of recombinant *Leishmania* antigens and their polyclonal antibodies

LACK, TSA and LmSTI1 cDNAs were ligated into the *Escherichia coli* protein expression vector pET32a (Novagen, Darmstadt, Germany), for expression as proteins fused to an N-terminal histidine tag. Competent BL21 cells were transformed with the plasmids, and 1-L cell cultures were induced to express the recombinant proteins at mid-log phase of growth (OD_600_ = 0.2) by the addition of 1 mM isopropyl β-d-1-thiogalactopyranoside. After 3 h, the bacteria were collected and washed with PBS. The bacteria were lysed in lysis buffer (1% Triton X-100, 50 mM Tris-HCl [pH7.5], 50 mM NaCl, 1 mM EDTA, 1 mM dithiothreitol) and centrifuged at 15,000 × *g* for 30 min. Recombinant TSA was mainly produced in the soluble fraction and recombinant LACK and LmSTI1 in the insoluble fraction. The TSA protein in the supernatant was affinity purified with a Ni–nitrilotriacetic acid (NTA) resin column (GE Healthcare, Amersham, UK), according to the manufacturer’s protocol, with the AKTA Prime FPLC chromatography system (GE Healthcare). LACK and LmSTI1 in the insoluble pellets were lysed with 6 M guanidine-HCl, applied to NTA resin column, and eluted with AKTA Prime FPLC chromatography system according to the manufacturer’s instructions. Each antigen (100 μg) was mixed with RIBI adjuvant (Corixa Corporation, Seattle, WA, USA) and used to immunize rabbits twice at a 1-month interval. Sera were collected 55 days after the first immunization.

### Immunoblotting analysis

B95a cells infected with each of the CDVs were washed once with PBS and lysed with 1% Triton X-100 in 10 mM Tris–HCl (pH 7.5), 5 mM EDTA, 1 mM dithiothreitol, 0.25 mM PMSF. Each sample was separated by 10% SDS-PAGE and blotted onto Immobilon-P membrane (Millipore, Billerica, MA, USA). After the membranes were blocked with PBS containing 4% BLOCK ACE Reagent (DS Pharma Biomedical, Osaka, Japan) and 0.05% Tween 20, they were incubated with rabbit anti-LACK antibody, rabbit anti-TSA antibody, rabbit anti-LmSTI1 antibody (described above), or rabbit anti-N protein antibody for 1 h at room temperature. After the membranes were washed, they were incubated with goat anti-rabbit IgG antibody conjugated with horseradish peroxidase (Dako Cytomation, Glostrup, Denmark) and then treated with ECL western blotting detection reagent (GE Healthcare). The reaction was visualized on an LAS 1000 Image Analyzer (Fujifilm, Tokyo, Japan).

### Growth kinetic analysis

Monolayers of B95a cells in a 24-well plate were infected with virus at a multiplicity of infection (MOI) of 0.01. The infected cells and medium were harvested daily, frozen and thawed three times, and centrifuged at 15,000 × *g* for 10 min. Virus titers of the supernatants were determined as TCID_50_ values using standard methods.

### Ethics statement

All animal experiments followed the laws of Japan: The Law for the Humane Treatment and Management of Animals (Act No. 105 of October 1, 1973) and the Law Concerning the Conservation and Sustainable Use of Biological Diversity through Regulations on the Use of Living Modified Organisms (Act No. 97 of June 18, 2003). All animal experiments were approved by the Animal Experiment Committee at the University of Tokyo (approval numbers: 13–56, 18–23, A11-41), and were performed in accordance with the Regulations for Animal Care and Use of the University of Tokyo, which were developed under the two laws stated above and nine guidelines including Fundamental Guidelines for Proper Conduct of Animal Experiment and Related Activities in Academic Research Institutions under the jurisdiction of the Ministry of Education, Culture, Sports, Science and Technology, and Standards Relating to the Care and Management of Laboratory Animals and Relief of Pain under the jurisdiction of the Ministry of the Environment. All surgery was performed under anesthesia with Dormicum and Domitor. All efforts were made to minimize animal suffering. At the end of the experiments, the dogs were euthanized by exsanguination under anesthesia induced with ketamine–xylazine.

### Experimental animals

Female beagle puppies, 5 weeks of age and confirmed free of CDV infection by an anti-CDV antibody enzyme-linked immunosorbent assay (ELISA), were purchased from Nihon Nosan (Yokohama, Japan). The dogs were group-housed in cages with ample space for exercise. The groups of dogs were kept in strict isolation to prevent viral cross-contamination during the course of all experiments.

### Immunization and challenge with virulent CDV

The animal experiments were conducted using two dogs per group. The dogs were subcutaneously inoculated with 500 μl of rCDV–LACK (titer of 10^4.5^ TCID_50_ per ml) on days 0 and 14. Unimmunized mock-treated control dogs were inoculated with 500 μl of phosphate-buffered saline (PBS). The dogs were challenged intracerebrally with 500 μl of 10% brain homogenate infected with CDV strain Snyder Hill (described above in the ‘Cells, viruses and parasite’ section of the Methods), 21 days after the first immunization. After challenge, the rectal temperatures, leukocyte counts, and clinical signs of the dogs were recorded daily for 7 days.

### Immunization and challenge with *L*. *major*

The animal experiments were conducted using two dogs per group. The dogs were subcutaneously inoculated with 500 μl of parental CDV-Yanaka, rCDV–LACK, rCDV–TSA, rCDV–LmSTI1 (all titers 10^4.5^ TCID_50_ per ml), or a cocktail of three rCDVs (500 μl each) on days 0 and 14. Unimmunized mock-treated control dogs were inoculated with 500 μl of PBS. The dogs’ body temperatures, bodyweights, and clinical signs were checked daily for 21 days. Their leukocyte counts were checked 0, 7, 14 and 21 days after the first vaccination. The dogs were inoculated intradermally (in the ears) with infective promastigotes of *L*. *major* PM2 (5 × 10^7^ parasites per spot) 42 days after the first vaccination. Every week after challenge, the sizes of the nodules on the ears were measured (mm^2^) with calipers.

### ELISA

The production of antibodies against CDV, LACK, TSA and LmSTI1 in dog sera were determined with an ELISA. When anti-CDV antibodies were checked, the extracts of either the CDV-Yanaka-infected B95a cells or mock infected cells were used. Recombinant LACK, TSA and LmSTI1 described above were utilized for the detection of respective antibodies. ELISA was performed using 96-well plates with a standard method. In brief, the plates were consecutively incubated with various dilutions of dog sera and sheep anti-dog IgG conjugated with HRP (Cappel Lab., Cochranville, PA, USA) and then with the ELISA substrate (Bio Rad, Hercules, CA, USA), and optical density values at 492 nm (OD_492_) were measured.

### Detection of *L*. *major*

At 74 days after challenge, the dogs were euthanized, and the ears, spleen, bone marrow, liver and parotid lymph node were collected and subjected to a detection test. In brief, these tissues were suspended in C-M199 medium, and an aliquot of the suspension was combined with blood agar plates and incubated at 26°C for 7 days. The presence of parasites was observed using an inverted microscope.

## Results

### Rescue and growth characteristics of rCDVs expressing *Leishmania* genes

We first generated rCDV-Yanaka expressing *Leishmania* antigens. We selected three protein antigens: LACK, TSA and LmTSI1. The *LACK*, *TSA* or *LmTSI1* gene was inserted into the cDNA clone of the CDV-Yanaka genome between the N and P genes (Fig 1A). The plasmids were then used in our CDV rescue system [21]. Three days after HEK293 cells were overlain with B95a cells, a typical cytopathic effect was observed.

Protein expression by the rescued viruses was confirmed with immunoblotting (Fig 1B), indicating that the recombinant viruses were successfully rescued. The rescued viruses were designated rCDV-LACK, rCDV-TSA and rCDV-LmSTI1, respectively. These rCDVs showed similar viral growth to the parental CDV-Yanaka strain (Fig 1C).

### Vaccine efficacy against lethal CDV challenge in dogs

We previously demonstrated that the CDV-Yanaka strain is avirulent and confers protective immunity against lethal doses of virulent CDVs in dogs [20]. To investigate whether the insertion of a foreign gene into the CDV genome affected the original features of CDV-Yanaka, dogs were inoculated with each rCDV and monitored daily. Similarly to dogs inoculated with the parental CDV-Yanaka strain, dogs inoculated with rCDV showed no clinical signs of CD, including leucopenia or pyrexia, demonstrating that the rCDVs are safe for dogs, even when expressing *Leishmania* antigens. The antibody responses were analyzed by ELISA. An increase of anti-CDV antibodies was found in all dogs’ sera on Day 21, while interestingly, no LACK antibodies were observed for more than 50 days after the first vaccination by ELISA.

**Fig 2:**
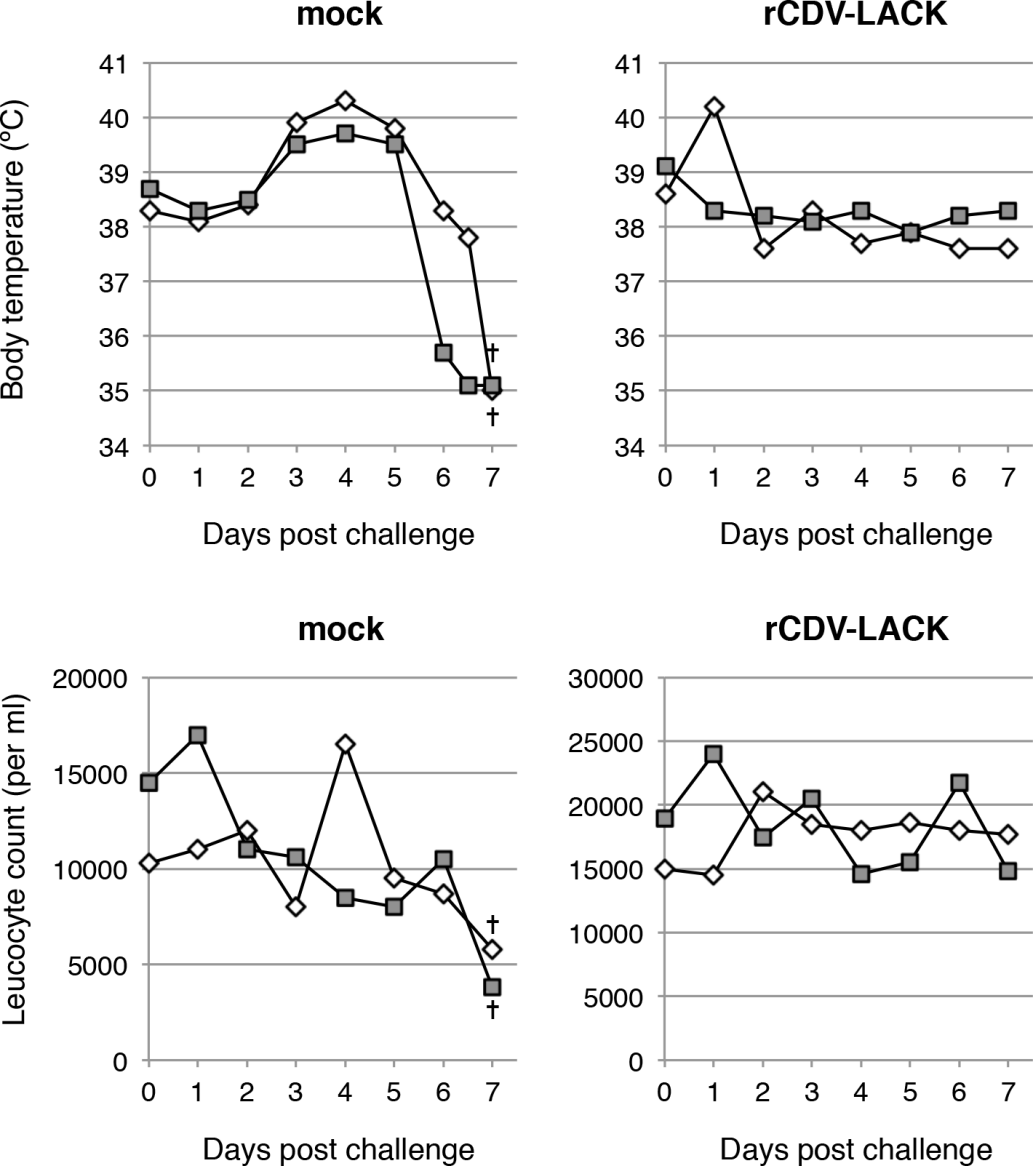
Changes in body temperatures and leukocyte counts after virulent CDV challenge. The body temperatures and leucocyte counts of mock- and rCDV-LACK-immunized dogs after challenge with the virulent Snyder Hill strain of CDV were measured daily. At 7 days after challenge, the mock-treated dogs were in a moribund state and were euthanized (†). Each symbol represents one animal.

To confirm the protective effects of the rCDVs against virulent CDV, dogs inoculated with PBS or rCDV-LACK were challenged with the virulent CDV strain, Snyder Hill. The mock-inoculated control dogs showed severe pyrexia and leucopenia, and the body temperatures of the dogs rapidly fell below 35°C (Fig 2). They were euthanized 7 days after challenge in a moribund state. In contrast, the rCDV-LACK-vaccinated dogs showed no specific clinical signs of distemper (Fig 2). This result indicated that the expression of a foreign gene does not affect the protective immunity against CDV conferred by CDV-Yanaka.

### *L*. *major* challenge in dogs

**Fig 3:**
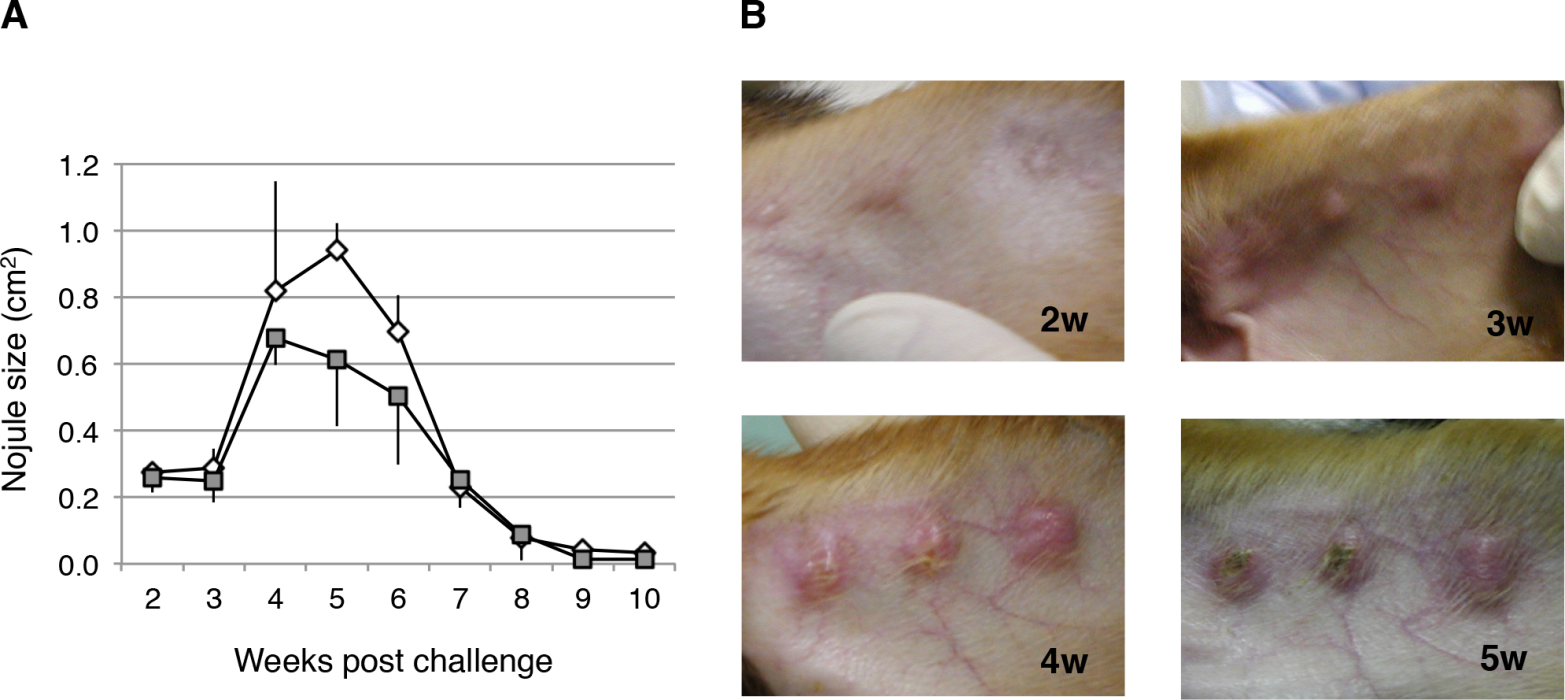
Time course of skin lesion development in dogs infected with *L*. *major*. (A) Beagle dogs were infected intradermally (in the ears) with 5 × 10^7^ infective promastigotes of *L*. *major* per spot, and the lesion sizes were measured weekly. Parasite growth was evaluated as nodule size. Three independent spots per dog were determined and followed-up. Data are shown as means ± SEM, and the error bars reflect the three inoculated spots. (B) Images of lesions at 2 to 5 weeks after infection.

Next we attempted to establish experimental models for canine cutaneous leishmaniasis. To evaluate the utility in this model of *L*. *major*, a major species responsible for cutaneous leishmaniasis, dogs were inoculated intradermally (three spots in the ears) with infective promastigotes (5 × 10^7^ per spot) of the parasite. As shown in Fig 3, the parasites proliferated at the sites of inoculation, and formed nodules in the skin lesions. The nodules first appeared in the second week and ulcers were observed in the third week (Fig 3B). The nodules became enlarged, reaching their maximum size in the fourth or fifth week, with typical crater-like lesions, and then regressed (Fig 3A).

### Protective effect of rCDVs expressing *Leishmania* antigens against *L*. *major* challenge

**Fig 4:**
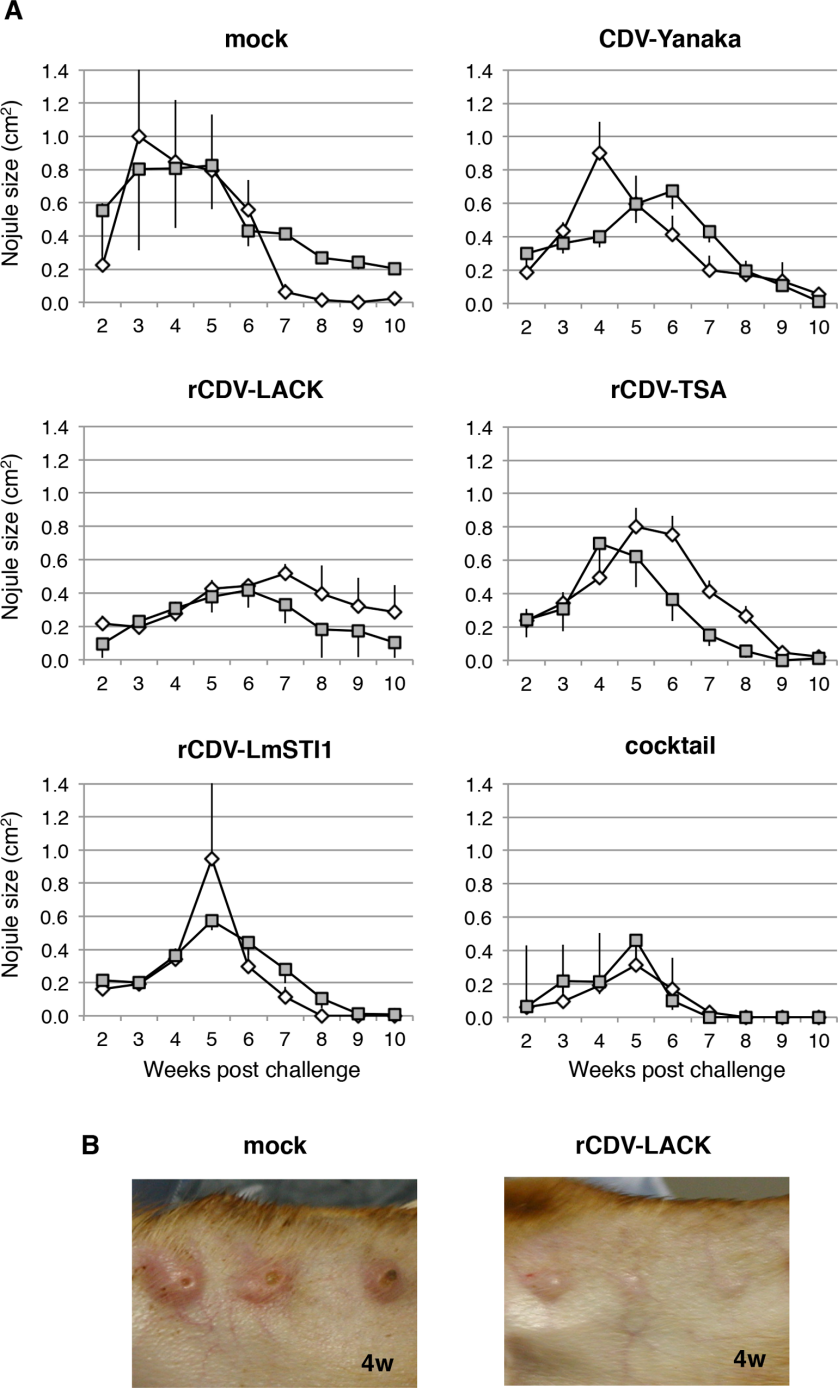
Protective efficacy of immunization with rCDV-LACK, rCDV-TSA and rCDV-LmSTI1 against *L*. *major* challenge. (A) Dogs were inoculated intradermally (in the ears) with 5 × 10^7^ infective promastigotes of *L*. *major* per spot, 42 days after their first vaccination with the indicated inoculum. Parasite growth was evaluated every week as nodule size. Three independent spots were inoculated per dog and followed-up. Data are shown as the mean ± SEM, and the error bars reflect the three inoculated spots. (B) Photographs of the nodules on the ears of mock- and rCDV-LACK-immunized dogs in the fourth week after *L*. *major* challenge.

Using this animal model, we evaluated the efficacy of the rCDVs as vaccines against *L*. *major*. Two dogs each were immunized twice with PBS (mock), parental CDV-Yanaka, or each rCDV. An increase in anti-CDV antibodies was found in all dogs, while anti-TSA and anti-LmSTI1 antibodies levels were not as readily detectable as anti-LACK antibody. Four weeks after the second immunization, *L*. *major* was challenged, as described above. As shown in Fig 4A, nodule formation in the mock-inoculated dogs displayed reproducible progression (Fig 3). Nodule formation was slightly slower in the dogs immunized with parental CDV-Yanaka, but the sizes of the nodules were similar to those in the mock-immunized dogs. Interestingly, in the rCDV-LACK-vaccinated dogs, the nodules were smaller than those in the mock-immunized dogs, particularly up to 5 weeks after challenge (Fig 4A). Furthermore, none of the nodules in the rCDV-LACK-vaccinated dogs were ulcerated (Fig 4B). This result indicated that vaccination with rCDV-LACK conferred marked protective immunity, effectively suppressing the proliferation of *L*. *major* at an early stage of infection. Dogs immunized with rCDV-TSA or rCDV-LmSTI1 showed similar nodule growth to that observed in the CDV-Yanaka-immunized dogs (Fig 4A), suggesting that the expression of TSA or LmSTI1 alone produced only a weak immunogenic effect.

Therefore, we also vaccinated dogs with a cocktail of rCDV-LACK, rCDV-TSA, and rCDV-LmTSI1. The nodules in the cocktail-immunized dogs were significantly smaller than those in the single-vaccine-immunized dogs (Fig 4A). In particular, the nodules in the cocktail-immunized dogs had decreased rapidly in size after the sixth week of challenge. This suggested that the period of low-level parasite proliferation observed in the rCDV-LACK-vaccinated dogs was significantly suppressed in the cocktail-vaccinated dogs. All dogs were euthanized at the 10^th^ week after the challenge. The tissues of ears, spleen, bone marrow, liver and the parotid lymph node were collected and the parasites were detected. As shown in Table 1, no parasite was detected in these tissues of the unvaccinated dogs. By contrast, in the CDV-LACK vaccinated dogs, *L*. *major* was detected in several tissues. These results showed that CDV-LACK vaccination suppressed proliferation of parasites, and caused a delay in parasite clearance. In cocktail-immunized dogs, no parasite was detected. Therefore, the cocktail immunization suppressed the proliferation of *L*. *major* at all stages of infection more effectively than immunization with the single rCDV-based vaccines.

**Table 1:** Detection of *L*. *major* at the 10th week post challenge.

Immunized virus	Tissue type				
	Ear	Parotid lymph node	Spleen	Bone marrow	Liver
Mock	-	-	-	-	-
	-	-	-	-	-
rCDV-LACK	-	**+**	-	-	-
	**+**	**+**	-	-	-
Cocktail	-	-	-	-	-
	-	-	-	-	-

-: not detected

+: detected

This is the first report of a dog model of cutaneous leishmaniasis that is appropriate for testing vaccines. We also demonstrated that rCDVs expressing *Leishmania* antigens confer protective immunity against both virulent CDV and *L*. *major* challenge in dogs.

## Discussion

We previously examined the CDV-Yanaka strain as a potential novel live vaccine against recently prevalent CDV strains. In addition, we also previously established a reverse genetics system for the Yanaka strain [21], and the data presented here show that CDV-Yanaka is a safe and effective viral vector (Fig 2). The technique described here can simultaneously induce immunity against CDV and other pathogens.

Although visceral leishmaniasis has been studied extensively in dogs and various models have been described [30–35], little is known about canine cutaneous leishmaniasis, and only experimental infections with *L*. *(Viannia) braziliensis* [10] and *L*. *mexicana* [11] have been reported. In the present study, we presented an animal model of experimental infection with *L*. *major* in beagle dogs. The infection of dogs with *L*. *major* caused typical ulcerated skin lesions to develop, with similar sizes in all dogs and a rapid onset 3 to 5 weeks after infection (Figs 3 and 4). This infection model is highly reproducible. The progression of the lesions of *L*. *major* was similar to those of *L*. *mexicana* [11]. Therefore, challenging dogs with *L*. *major* generates a suitable animal model of cutaneous leishmaniasis. In particular, the progression of nodules slowed at about 10 weeks in control dogs, which is desirable compared with canine visceral leishmaniasis which usually takes over 1 year.

Based on this information, we evaluated the utility of our rCDVs as effective polyvalent candidate vaccines against CDV and *L*. *major* infections. The results of this study indicated that vaccination with rCDV-LACK markedly reduced the nodule size after *L*. *major* challenge, particularly in the early phase of infection (Fig 4). Previous studies of *L*. *major* infection in a mouse model demonstrated that the protective efficacy of LACK is mainly observed in cutaneous leishmaniasis [6–9], so we consider our results to be reproducible. Studies with a mouse model also demonstrated that plasmid DNA encoding TSA or LmSTI1 partially or markedly protected the mice against *L*. *major* challenge, respectively [6–9]. In contrast, rCDV-TSA and rCDV-LmSTI1 showed little immunogenic efficacy in the present study (Fig 4A), suggesting that TSA and LmSTI1 alone are weakly immunogenic in dogs. In contrast, combined immunization with rCDV-LACK, rCDV-TSA, and rCDV-LmSTI1 produced a more effective result against *L*. *major* challenge than immunization with each construct alone (Fig 4A). Previous studies have demonstrated that combinations of TSA and LmSTI1 proteins conferred strong protective immunity against *L*. *major* challenge in mice and monkeys [23]. Immunization with a fusion protein, designated “Leish 111”, composed of TSA, LmSTI1 and LeIF (*Leishmania* elongation initiation factor), or its derivative Leish 110, together with an adjuvant, conferred significant protection against *Leishmania* challenge, producing smaller lesions in mice [6–9]. These results strongly suggest that a cocktail of multiple antigens confers more effective immunity throughout the life cycle of *Leishmania* than single antigens. In particular, the cocktail vaccine reduced the time period between challenge and cure compared with that achieved with the rCDV-LACK vaccine (Fig 4A). The reduction in nodule size may be mainly attributable to rCDV-LACK, and the shortened duration of the disease to rCDV-TSA and/or rCDV-LmSTI1.

Many previous reports have indicated that the interferon γ (IFN-γ)-dominant Th1 response phenotype was associated with protection against *L*. *major* infection, whereas the Th2 response phenotype (with IL-4) was associated with susceptibility to, or aggravation of, the disease in a murine model [36–39]. Our present study has demonstrated that rCDV, and particularly a cocktail of three rCDVs, induced marked protective immunity against *L*. *major* challenge, although slight but apparent proliferation of the parasite occurred in the early stage of infection in the cocktail-vaccinated dogs. This phenomenon suggests that the Th1 immune response is insufficient to completely suppress parasite proliferation. The role of IL-12 as a cytokine adjuvant to improve the efficacy of *Leishmania* vaccines has been well studied. IL-12 is a potent activator of IFN-γ production in natural killer and T cells and promotes the development of Th1 responses [40,41]. Many reports have demonstrated that the coadministration of IL-12 with recombinant proteins, including an LmSTI1/TSA cocktail or DNA vaccines, results in robust, long-lasting protection against *Leishmania* challenge in animals [6–9]. However, IL-12 is encoded by two separate genes, requiring it to be expressed simultaneously in one cell for formation of the heterodimer. However, it has recently been reported that IL-18 also augments the IFN-γ-inducing capacity of vaccines, independently of IL-12 [42]. IL-18 has also been tested as a vaccine adjuvant, and enhanced the efficiencies of various vaccines in mammals [43–46]. Recently, we generated a rCDV that secretes bioactive canine IL-18 and induces IFN-γ production by canine peripheral blood mononuclear cells [47]. This recombinant virus can potentially be used as an immunoadjuvant *in vivo*.

We propose that a combination of rCDV-based vaccines expressing different antigens with different effects on the immune response, has utility as a polyvalent vaccine for the prevention of leishmaniasis epidemics by inhibiting the transmission of the parasites through dogs.
